# Supplementary material for: Heat adaptation of phage T7 under an extended genetic code
Source: Virus Evol. 2021 Dec 1;7(2):veab100. doi: 10.1093/ve/veab100 (PMC8923235; doi:10.1093/ve/veab100)
Supplement: veab100_Supp [file veab100_supp.zip › Sequencing_Analysis.zip!/Sequencing_Analysis/A2/evidence/JC_SIDE_1_55.html]

BRESEQ :: Evidence


| New junction evidence | | | | | | | | | | | |
| --- | --- | --- | --- | --- | --- | --- | --- | --- | --- | --- | --- |
|  | | seq id | position | reads (cov) | reads (cov) | score | skew | freq | annotation | gene | product |
| \* | ? | NC\_001604.1 | = 16604 | 373 (0.820) | 30 (0.070) | 26/590 | 12.4 | 6.9% | intergenic (–/–) | *–/–* | –/– |
|  | ? | NC\_001604.1 | 17028 = | 438 (0.980) |  |  |  |  | intergenic (–/–) | *–/–* | –/– |
| Rejected: Coverage evenness skew score above cutoff. | | | | | | | | | | | |
| Rejected: Frequency below/above cutoff threshold. | | | | | | | | | | | |

Only 100 of 375 total aligned reads displayed.  

`GGGTACATGATGAAATCCAAGTAGGCTGCCGTACCGAAGAGATTGCTCAGGTGGTCATTGAGACCGCACAAGAAGCGATGCGCTGGGTTGGAGACCACTGGAACTTCCGGTGTCTTCTGGATACCGAAGGTAAGATGGGTCCTAATTGGGCGATTTGCCACTGATACAGGAGGCTACTCATGAACGAAAGACACTTAACAGGTGCTGCTTCTGAAATGCTAGTAGCCTACAAATTTACCAAAGCTGGGTACACTGTCTATTACCCTATGCTGACTCAGAGTAAAGAGGACTTGGTTGTATGTAAGGATGGTAAATTTAGTAAGGTTCAGGTTAAAACAGCCACAACGGTTCAAACCAACACAGGAGATGCCAAGCAGGTTAGGCTAGGTGGATGCGGTAGGTCCGAATATAAGGATGGAGACTTTGACATTCTTGCGGTTGTGGTTGACGAAGATGTGCTTATTTTCACATGGGACGAAGTAAAAGGTAAGACATCCATGTGTGTCGGCAAGAGAAACAAAGGCATAAAACTATAGGAGAAATTATTATGGCTATGACAAAGAAATTTAAAGTGTCCTTCGACGTTACCGCAAAGATGT  >  NC_001604.1/16304‑16902`  

                                                                                                                                                                                                                                                                                                            `|`                                                                                                                                                                                                                                                                                                            
`gggTACAAGATGAAAGCCAAGTAGGCTGCCGTACCGAAGAGATTGCTCAGGTGGTCATTGAGCCCGCACAAGAAGCGATGCGCGGGGTTGGAGACCACTGGAACTTCCGGTGTCTTCTTGATAACGAAGGTAAGATGGGTCCTAATTGGGCGATTTGCCACTGATACAGGAGGCTACTCATGAACGAAAGACACGTAACAGGTGCTGCTTCTGAAATGCTAGTAGCCTACAAATTTACCAAAGCTGGGTACACTGTCTATTACCCTATGCTGACTCAGAGTAAAGAGGACTTGGTtgtatg                                                                                                                                                                                                                                                                                                            <  1:251005/301‑1 (MQ=255)`  
    `aCATGATGAAATCAAAGTAGGCTGCCGTACCGAAGAGATTGCTCAGGTGGTCATTGAGACCGCACAAGAAGCGATGCGCTGGGTTGGAGACCACTGGAACTTACGGTGTCTTCTGGATACCGAAGGTAAGATGGGTCCTAATTGGGAGATTTGCAACTGATACAGGAGGCTACTCATGAACGAAAGACACTTAACAGGTGCTGCTTCTGAAATGCTAGTAGCCTACAAATTTACCAAAGCTGGGTACACTGTCTATTACCCTATGCTGACTCAGAGTAAAGAGGACTTGGTTGTATGTAAg                                                                                                                                                                                                                                                                                                        <  1:440139/301‑1 (MQ=255)`  
     `cATGATGAAATACAAGTAGGCTGCCGTACCGAAGAGAGTGCTCAGGTGGTCATTGAGACCGCACAAGAAGCGATGCGCTGGGTTGGAGACCACTGGAACTTCCGGTGTCTTCTGGATACCGAAGGTAAGATGGGTCCTAATTGGGCGATTTGCCACTGATACAGGAGGCTACTCATGAACGAAAGACACTTAACAGGTGCTGCTTCTGAAATGCTAGTAGCCTACAAATTTACCAAAGCTGGGTACACTGTCTATTACCCTATGCTGACTCAGAGTAAAGAGGACTTGGTTGTATGTAAgg                                                                                                                                                                                                                                                                                                       <  1:362691/301‑1 (MQ=255)`  
              `aTCCAAGTAGTCTGCCGTACAGAAGAGATTGCTCAGGTGGTCATTGAGACCGCACAAGAAGCGATGCGCTGGGTTGGAGACCACTGGAACTTCCGGTGTCTTCTGGATACCGAAGGTAAGATGGGTCCTAATTGGGCGATTTGCCACTGATACAGGAGGCTACTCATGAACGAAAGACACTTAACAGGTGCTGCTTCTGAAATGCTAGTAGCCTACAAATTTACCAAAGCTGGGTACACTGTCTATTACCCTATGCTGACTCAGAGTAAAGAGGACTTGGTTGTATGTAAGGATGGTAAAt                                                                                                                                                                                                                                                                                              >  1:810605/1‑301 (MQ=255)`  
               `tCCAAGTAGGCTGCCGTACCGAAGAGATTGCTCCGGTGGTCATTGCGACCGCACAAGAAGCTATGCGCTGGGTTGGAGACCACTGGAACTTCCGGTGTCTTCTGGATACCGAAGGTAAGATGGGTCCTAATTGGGCGATTTGCCACTGATACAGGAGGCTACTCATGAACGACAGACACTTAACAGGTGCTGCTTCTGAAATGCTAGTAGCCTACATATTTACCAAAGCTGGGTACACTGTCTATTACCCTATGCTGACTCAGAGTAAAGAGGACTTGGTTGTATGTATGGATGGTAAAtt                                                                                                                                                                                                                                                                                             >  1:173769/1‑301 (MQ=255)`  
                `ccAAGTAGGCTGCCGTACCGAAGAGATTGCTCAGGTGGTCATTGAGACCGCACAAGAAGCGATGCGCTGGGTTGGAGACCACTGGAACTTCCGGTATCTTCTGGATACCGAAGGTAAGATGGGTCCTAATTGGGCGATTTGCCACTGATACAGGAGGCTACTCATGAACGAAAGACACTTAACAGGTGCTGCTTCTGAAATGCTAGTAGCATACAAATTTACCAAAGCTGGGTAAACTGTCTATTACCCTATGCTGACTCAGAGTAAAGAGGACTTGGTTGTATGTAAGGATGGTAAAttt                                                                                                                                                                                                                                                                                            >  1:645947/1‑301 (MQ=255)`  
                              `gagtctcgtgggctcggagatgtgtataagagaCAGCACAAGAAGCGATGCGCTGGGTTGGAGACCACTGGAACTTCCGGTGTCTTCTGGATACCGAAGGTAAGATGGGTCCTAATTGGGCGATTTGCCACTGATACAGGAGGCTACTCATGAACGAAAGACACTTAACAGGTGCTGCTTCTGAAATGCTAGTAGCCTACAAATTTACCAAAGCTTGGTACACTGTCTATTACCCTATGCTGACTCAGAGTAAAGAGGACTTGGTTGTATGTAAGGATGGTAAATTTAGTAAGGTTCAGGt                                                                                                                                                                                                                                                                              <  1:397556/272‑1 (MQ=255)`  
                              `gTACCGAAGAGATTGCTCAGGTGGTCATTGAGACCGCACAAGAAGCGATGCGCTGGGTTGGAGACCACTGGAACTTCCGGTGTCTTCTGGATACCGAAGGTAAGATGGGTCCTAATTGGGCGATTTGCCACTGATACAGGAGGCTACTCATGAACGAAAGACACTTAACAGGTGCTGCTTCTGAAATGCTAGTAGCCTACAAATTTACCAAAGCTTGGTACACTGTCTATTACCCTATGCTGACTCAGAGTAAAGAGGACTTGGTTGTATGTAAGGATGGTAAATTTAGTAAGGTTCAGGt                                                                                                                                                                                                                                                                              >  1:156202/1‑301 (MQ=255)`  
                                    `cgtgggctcggagatgtgtataagagaCAGCACAAGAAGCGATGCGCTGGGTTGGAGACCACTGTAACTTCCGGTGTCTTCTGGATACCGAAGGTAAGATGGGTCCTAATTGGGCGATTTGCCACTGATACAGGAGGCTACTCATGAACGAAAGACACTTAACAGGTGCTGCTTCTGAAATGCTAGTAGCCTACAAATTTACCAAAGCTGGGTACACTGTCTATTACCCTATGCTGACTCAGAGTAAAGAGGACTTGGTTGTATGTAAGGATGGTAAATTTAGTAAGGTTCAGGTTAAAAc                                                                                                                                                                                                                                                                        <  1:270393/278‑1 (MQ=255)`  
                                    `aaGAGATTGCTCAGGTGGTCATTGGGACCGCACAAGAAGCGATGCGCTGGGTTGGAGACCACTGGAACTTCCGGTGTCTTCTGGATACCGAAGGTAAGATGGGTCCTAATTGGGCGATTTGCCACTGATACAGGAGGCTACTCATGAACGAAAGACACTTAACAGGTGCTGCTTCTGAAATGCTAGTAGCCTACAAATTTACCAAAGCTGGGTACACTGTCTATTACCCTATGCTGACTCAGAGTAAAGAGGACTTGGTTGTATGTAAGGATGGTAAATTTAGTAAGGTTCAGGTTAAAAc                                                                                                                                                                                                                                                                        <  1:130831/301‑1 (MQ=255)`  
                                    `aaGAGATTGCTCAGGTGGTCATTGAGACCGCACAAGAAGCGATGCGCTGGGTTGGAGACCACTGGAACTTCCGGTGTCTTCTGGATACCGAAGGTAAGATGGGTCCTAATTGGGCGATTTGCCACTGATACAGGAGGCTACTCATGAACGAAAGACACTTAACAGGTGCTGCTTCTGAAATGCTAGTAGCCTACAAATTTACCAAAGCTGGGTACACTGTCTATTACCCTATGCTGACTCAGAGTAAAGAGGACTTGGTTGTATGTAAGGATGGTAAATTTAGTAAGGTTCAGGTTAAAAc                                                                                                                                                                                                                                                                        <  1:777831/301‑1 (MQ=255)`  
                                    `aaGAGATTGCTCAGGTGGTCATTGAGACCGCACAAGAAGCGATGCGCTGGGTTGGAGACCACTGGAACTTCCGGTGTCTTCTGGATACCGAAGGTAAGATGGGTCCTAATTGGGCGATTTGCCACTGATACAGGAGGCTACTCATGAACGAAAGACACTTAACAGGTGCTGCTTCTGAAATGCTAGTAGCCTACAAATTTACCAAAGCTGGGTACACTGTCTATTACCCTATGCTGACTCAGAGTAAAGAGGACTTGGTTGTATGTAAGGATGGTAAATTTAGTAAGGTTCAGGTTAAAAc                                                                                                                                                                                                                                                                        <  1:303941/301‑1 (MQ=255)`  
                                            `gCTCAGGTGGTCATTGAGACCGCACAAGAAGCGATGCGATGGGTTGGAGACCACTGGAACTTCCGGTGTCTTCTGGATACCGAAGGTAAGATGGGTCCTAATTGGGCGATTGGCCACTGATACAGGAGGCTACTCATGAACGAAAGACACTTAACAGGTGCTGCTTCTGAAATGCGAGTAGACTACAAATTTACCAAAGCTGGGTACACTGTCTATTACCATATGCTGACACAGAGTAAAGAGGACTTGGTGGTATGTAAGGAGGGTAAATTTAGTAAGGTTCAGGTTAAAACAGCCACaa                                                                                                                                                                                                                                                                >  1:668790/1‑301 (MQ=255)`  
                                                 `ggtggtCATTGAGACCGCACAAGAAGCGATGCGCTGGGTTGGAGACCACTGGAACTTCCGGTGTCTTCTGGATACCGAAGGTAAGATGGGTCCTAATTGGGCGATTTGCCACTGATACAGGAGGCTACTCATGAACGAAAGACACTTAACAGGTGCTGCTTCTGAAATGCTAGTAGCCTACAAATTTACCAAAGCTGGGTACACTGTCTATTACCCTATGCTGACTCAGAGTAAAGAGGACTTGGTTGTATGTAAGGATGGTAAATTTAGTAAGGTTCAGGTTAAAACAGCCACAACGGtt                                                                                                                                                                                                                                                           >  1:580953/1‑301 (MQ=255)`  
                                                     `gtCATTTAGACCGCACAAGAAGCGATGCGCTGGGTTGGAGACCACTGGAACTTCCGGTGTCTTCTGGATACCGAAGGTAAGATGTGTCCTAATTGGGCGATTTGCCACTGATACAGGAGGCTACTCATGAACGAAAGACACTTAACAGGTGCTGCTTCTGAAATGCTAGTAGCCTACAAATTTACCAAAGCTGGGTACACTGTCTAATACCCTATGCTGACTCAGAGTAAAGAGGACTTGGTTGTATGTAAGGATGGTAAATTTAGTAAGGTTCAGGtctgtctcttatacacatctccga                                                                                                                                                                                                                                                       >  1:588018/1‑278 (MQ=255)`  
                                                      `tCATTGAGACCGCACAAGAAGCGATGCGCTGGGTTGGAGACCACTGGAACTTCCGGTGTCTTCTGGATACCGAAGGTAAGATGGGTCCTAATTGGGCGATTTGCCACTGATACAGGAGGCTACTCATGAACGAAAGACACTTAACAGGTGCTGCTTCTGAAATGCTAGTAGCCTACAAATTTACCAAAGCTGGGTACACTGTCTATTACCCTATGCTGACTCAGAGTAAAGAGGACTTGGTTGTATGTAAGGATGGTAAATTTAGTAAGGTTCAGGTTAAAACAGCCACAACGGTTCAAAc                                                                                                                                                                                                                                                      <  1:731080/301‑1 (MQ=255)`  
                                                       `aaTTGAGACCGAACAAGAAGCGATGCGCTGGGTTGGAGACCACTGGAACTTCCGGTGTCGTCTGGATACCGAAGGTAAGATGGGTCCTAATTGGGCGATTTGCCACTGATACAGGAGGCTACTCAGGAACGAAAGACACTTAACAGGTGCTGCTTCTGAAATGCTAGTAGCCTACAAATTTACCAAAGCTGGGTACACTGTCTATTACCATATGCTGACTCAGAGTAAAGAGGACGTGGTTGTATGTAAGGATGGTAAATTTAGTAAGGTTCAGGTTAAAACAGCCACAACGGTTCAAAcc                                                                                                                                                                                                                                                     <  1:231644/300‑1 (MQ=255)`  
                                                               `tgtgtataagagacaggcgcTGGGTTGGAGACCACTGGAACTTCCGGTGTCTTCTGGATACCGAAGGTAAGATGGGTCCTAATTGGGCGATTTGCCACTGATACAGGAGGCTACTCATGAACGAAAGACACTTAACAGGTGCTGCTTCTGAAATGCTAGTAGCCTACAAATTTACCAAAGCTGGGTACACTGTCTATTACCCTATGCTGACTCAGAGTAAAGAGGACTTGGTTGTATGTAAGGATGGTAAATTTAGTAAGGTTCAGGTTAAAACAGCCACAACGGTTCAAACCAACACAgg                                                                                                                                                                                                                                             <  1:640534/285‑1 (MQ=255)`  
                                                               `ggagatgtgtataagagacaggggTTTGAGACCACTGGAACTTCCGGTGTCTTCTGGATACCTAAGGTAAGATGTGTCCTAATTGGGCGATTTGCCACTGATACAGGAGGCTACTCATGAACGAAAGACACTTAACAGGTGCTGCTTCTGAAATGCTAGTATCCTACAAATTTACCAAAGCTGGGTACACTGTCTATTACCCTATGCTGACTCAGAGTAAAGAGGACTTGGTTGTATGTAAGGATGGTAAATTTAGTAAGGTTCAGGTTAAAACAGCCACAACGGTTCAAACCAACACAgg                                                                                                                                                                                                                                             <  1:103072/280‑1 (MQ=255)`  
                                                                  `gtcggagatgtgtataagagacaggAGACCACTGGAACGTCCGGTGTCTTCTGGATAACGAAGGTAAGATGGGTCCTAATTGGGCGATTTGCCACTGATACAGGAGGCTACTCATGAACGAAAGACACTTAACAGGTGCTGCTTCTGAAATGCTAGTAGCCTACAAATTTACCAAAGCTGGGTACACTGTCTATTACCCTATGCTGACTCAGAGTAAAGAGGACTTGGTTGTATGTAAGGATGGTAAATTTAGTAAGGTTCAGGTTAAAACAGCCACAACGGGGAAAACCAACACAGgaga                                                                                                                                                                                                                                          <  1:236942/278‑1 (MQ=255)`  
                                                                  `cacaAGAAGCGATGCGCTGGGTTGGAGACCACTGGAACTTCCGGTGTCTTCTGGATACCGAAGGTAAGATGGGTCCTAATTGGGCGATTTGCCACTGATACAGGAGGCTACTCATGAACGAAAGACACTTAACAGGTGCTGCTTCTGAAATGCTAGTAGCCTACAAATTTACCAAAGCTGGGTACACTGTCTATTACCCTATGCTGACTCATAGTAAAGAGGACTTGGTTGTATGTAAGGATGGTAAATTTAGTAAGGTTCAGGTTAAAACAGCCACAACTGTTCAAACCAACACAGtaga                                                                                                                                                                                                                                          >  1:159438/1‑301 (MQ=255)`  
                                                                      `ctcggagatgtgtataagagacagccACTGGAACTTCCGGTGTCTTCTGGATACCGAAGGTAAGATGGGTCCTAATTGGGCGATTTGCCACTGATACAGGAGGCTACTCATGAACGAAAGACACTTAACAGGTGCTGCTTCTGAAATGCTAGTAGCCTACAAATTTACCAAAGCTGGGTACACTGTCTATTACCCTATGCTGACTCAGAGTAAAGAGGACTTGGTTGTATGTAAGGATGGTAAATTTAGTAAGGTTCAGGTTAAAACAGCCACAACGGTTCAAACCAACACAGGAGATGcc                                                                                                                                                                                                                                      <  1:217133/277‑1 (MQ=255)`  
                                                                        `aagCGATGCGCTGGGTTGGAGACCACTGGAACTTCCGGTGTCTTCTGGATACCGAAGGTAAGATGGGTCCTAATTGGGCGATTTGCCACTGATACAGGAGGCTACTAATGAACGAAAGACACTTAACAGGTGCTGCTTCTGAAATGCTAGTAGCCTACAAATTTACCAAAGCTGGGTACACTGTCTATTACCATATGCTGACTCAGAGTAAAGAGGACTTGGTTGTATGTAAGGATGGTAAATTTAGTAAGGTTCAGGTTAAAACAGCCACAACGGTTCAAACCAACACAGGAGATGCCaa                                                                                                                                                                                                                                    >  1:680784/1‑301 (MQ=255)`  
                                                                              `ggcgcTCGGTTGGAGACCACTTGAACTTCCGGTGTCTTCTTGATACCGAAGGTAAGATTGGTCCTAATTGGGCTATTTGCCACTGATACAGGAGGCTACTCATGAACGAAAGACACTTAACAGGTGCTGCTTCTGAAATGCTAGTAGCCTACAAATTTACCAAAGCTGGGTACACTGTCTATTACCCTATGCTGACTCAGAGTAAAGAGGACTTGGTTGTATGTAAGGATGGTAAATTTAGTAAGGTTCAGGTTAAAACAGCCACAACGGTTCAAACCAACACAGGAGATGCCAAGCAGGt                                                                                                                                                                                                                              <  1:746945/300‑1 (MQ=255)`  
                                                                               `gcgcTGGGTTGGAGACCACTGGAACTTCCGGTGTCTTCTGGATACCGAAGGTAAGATGGGTCCTAATTGGGCGATTTGCCACTGATACAGGAGGCTACTCATGAACGAAAGACACTTAACAGGTGCTGCTTCTGAAATGCTAGTAGCCTACAAATTTACCAAAGCTGGGTACACTGTCTATTACCCTATGCTGACTCAGAGTAAAGAGGACTTGGTTGTATGTAAGGATGGTAAATTTAGTAAGGTTCAGGTTAAAACAGCCACAACGGTTCAAACCAACACAGGAGATGCCAAGCAGGtt                                                                                                                                                                                                                             <  1:795983/301‑1 (MQ=255)`  
                                                                                    `taagagacagccACTGGAACTTCCGGTGTCTTCTGGATACCGAAGGTAAGATGGGTCCTAATTGGGCGATTTGCCACTGATACAGGAGGATACTAATGAACGAAAGACACTTAACAGGTGCTGCTTCTGAAATGCTAGTAGCCTACAAATTTACCAAAGCTGGGTACACTGTCTATTACCCTATGCTGACTCAGAGTAAAGAGGACTTGGTTGTATGTAAGGATGGTAAATTTAGTAAGGTTCAGGTTAAAACAGCCACAACGGTTCAAACCAACACAGGAGATGCCAAGCAGGTTAGGCt                                                                                                                                                                                                                        <  1:141172/291‑1 (MQ=255)`  
                                                                                    `gggTTGGAGACCACTGGAACTTCCGGTGTCTTCTGGATACCGAAGGTAAGATGGGTCCTAATTGGGCGATTTGCCACTGATACAGGAGGCTACTCATGAACGAAAGACACTTAACAGGTGCTGCTTCTGAAATGCTAGTAGCCTACAAATTTACCAAAGCTGGGTACACTGTCTATTACCCTATGCTGACTCAGAGTAAAGAGGACTTGGTTGTATGTAAGGATGGTAAATTTAGTAAGGTTCAGGTTAAAACAGCCACAACGGTTCAAACCAACACAGGAGATGCCAAGCAGGTTAGGCt                                                                                                                                                                                                                        >  1:637532/1‑301 (MQ=255)`  
                                                                                         `ggAGACCACTAGCACTTCCAGTGACGTCGAGATAACGGAGATAAGATGGATCCAAATTGTGCGATTTGACACTGATTCAGGAAGCTAATCATGTACAAAAGTCACTTAACAGGTGCTGCTTCTGAAATGCTAGTAGCCTACTAATTTACCAAGGCTGGGTAAACTGTCTATTACCCTATGCTAACACAGAGTAAAAAGGACTTGGTTGTATGTAAGGATGGTAAATTTAGTAAGTTTCAGGTTAAAACAGCCACAACGGTTCAAACCAACACAGGAGATGCCAAGCAGGTTAGGCTAGGTg                                                                                                                                                                                                                   <  1:451642/301‑1 (MQ=255)`  
                                                                                              `ccACTGGAACTTCCGGTGTCTTCTGGATACCGAAGGTAAGATGGGTCCTAATTGGGCGATTTGCCACTGATACAGGAGGCTACTCATGAACGAAAGACACTTAACAGGTGCTGCTTCTGAAATGCTAGTAGCCTACAAATTTACCAAAGCTGGGTACACTGTCTATTACCCTATGCTGACTCAGAGTAAATAGGACTTGGTTGTATGTAAGGATGGTAAATTTAGTAAGGTTCATGTTAAAACAGCCACAACGGTTCAAACCAACACAGTAGATGCCAAGCAGGTTAGGCTAGGTGGATGc                                                                                                                                                                                                              >  1:575484/1‑301 (MQ=255)`  
                                                                                                      `aCTTCCGGTGTATTCTGGATACCGAAGGTAAGATGGGTCCTAATTGGGCGATTTGCCACTGATACAGGAGGCTACTCATGAACGAAAGACACTTAACAGGTGCTGCTTCTGAAATGCTAGTAGCCTACAAATTTACCAAAGCTGGGTACACTGTCTATTACCCTATGCTGACTCAGAGTAAAGAGGACTTGGTTGTATGTAAGGATGGTAAATTTAGTAAGGTTCAGGTTAAAACAGCCACAACGGTTCAAACCAACACAGGAGATGCCAAGCAGGTTAGGCTAGGTGGATGCGGTAGGTc                                                                                                                                                                                                      <  1:40234/301‑1 (MQ=255)`  
                                                                                                                 `cttctGGATACCGAAGGTAAGATGGGTCCTAATTGGGCGATTTGCCACTGATACAGGAGGCTACTCATGAACGAAAGACACTTAACAGGTGCTGCTTCTGAAATGCTAGTAGCCTACAAATTTACCAAAGCTGGGTACACTGTCTATTACCCTATGCTGACTCAGAGTAAAGAGGACTTGGTTGTATGTAAGGATGGTAAATTTAGTAAGGTTCAGGTTAAAACAGCCACAACGGTTCAAACCAACACAGGAGATGCCAAGCAGGTTAGGCTAGGTGGATGCGGTAGGTCCGAATATAAgg                                                                                                                                                                                           >  1:570637/1‑301 (MQ=255)`  
                                                                                                                  `aatgtgtataagagacagagagGGGTCCTAATTGGGCGATTTGCCACTGATACAGGAGGCTAAGCATGAACGAAAGAAACTTAACAGGTGCTGCTTCTGAAATGCTAGTAGCCTACAAATGTACCAAAGCTGGGTACACTGTCTATTACCCTATGCTGACTCAGAGTAAAGAGGAATTGGTTGTAGGTAAGGATGGTAAATTGAGTAAGGTTAAGGTTAAAACAGCAACAACGGTTCAAACAAACACAGGAGATGCCAAGCAGGTTAGGCTAGGTGGATGCGGTAGGTCCGAATATAAGGa                                                                                                                                                                                          <  1:498754/283‑1 (MQ=255)`  
                                                                                                                   `tctTGATACCGAAGGTAAGATGGGTCCTAATTGGGCGATTTGCCACTGATACAGGAGGCTACTCATGAACGAAAGACACTTAACAGGTGCTGCTTCTGAAATGCTAGTAGCCTACAAATTTACCAAAGCTGGGTACACTGTCTATTACCCTATGCTGACTCAGAGTAAAGAGGACTTGGTTGTATGTAAGGATGGTAAATTTAGTAAGGTTCAGGTTAAAACAGCCACAACGGTTCAAACCAACACAGGAGATGCCAAGCAGGTTAGGCTAGGTGGCTgtctcttatacacatctccgagc                                                                                                                                                                                         >  1:616125/1‑279 (MQ=255)`  
                                                                                                                   `tctGGATACCGAAGGTAAGATTGGTCCTAATTGGGCGATTTGCCACTGATACAGGAGGCTACCCATGAACGAAAGACACTTAACAGGTGCTGCTTCTGAAATGCTAGTAGCCTACAAATTTACCAAAGCTGGGTACACTGTCTATTACCCTATGCTGACTCAGAGTAAAGAGGACTTGGTTGTATGTAAGGATGGTAAATTTAGTAAGGTTCAGGTTAAAACAGCCACAACGGTTCAAACCACAACAGGAAATGCCAAGCAGGTTAGGCTAGGTGGATGCGATAGGTCCGAATATAATGAt                                                                                                                                                                                         >  1:694533/1‑301 (MQ=255)`  
                                                                                                                       `gATACCGAAGGTAAGATGGTTCCTAATTGGGCGATTTGCCACTGATACAGGAGGCTACTCATGAACGAAAGACACTTAACAGGTGCTGCTTCTGAAATGCTAGTAGCCTACAAATTTACCAAAGCTGGGTACACTGTCTATTACCCTATGCTGATTCAGAGTAAAGAGGACTTGGTTGTATGTAAGGATGGTAAATTTAGTAAGGTTCAGGTTAAAACAGCCACAACGGTTCAAACCAACACAGGAGATGCCAAGCAGGTTAGGCTAGGTTGATGCGGTAGGTCCGAATATAAGGATGgag                                                                                                                                                                                     <  1:686632/301‑1 (MQ=255)`  
                                                                                                                       `gATACCGAAGGTAAGATGGGTCCTAATTGGGCGATTTGCCACTGATACAGGAGGCTACTCATGAACGAAAGACACTTAACAGGTGCTGCTTCTGAAATGCTAGTAGCCTACAAATTTACCAAAGCTGGGTACACTGTCTATTACCCTATGCTGACTCAGAGTAAAGAGGACTTGGTTGTATGTAAGGATGGTAAATTTAGTAAGGTTCAGGTTAAAACAGCCACAACGGTTCAAACCAACACAGGAGATGCCAAGCAGGTTAGGCTAGGTGGATGCGGTAGGTCCGAATATAAGGATGgag                                                                                                                                                                                     <  1:2827/301‑1 (MQ=255)`  
                                                                                                                        `aTACCGAAGGTAAGATGGGTCCTAATTGGGCGATTTGCCACTGATACAGGAGGCTACTCATGAACGAAAGACACTTAACAGGTGCTGCTTCTGAAATGCTAGTAGCCTACAAATTTACCAAAGCTGGGTACACTGTCTATTACCCTATGCTGACTCAGAGTAAAGAGGACTTGGTTGTATGTAAGGATGGTAAATTTAGTAAGGTTCAGGTTAAAACAGCAACAACGGTTCAAACCAACACAGGAGATGCCAAGCAGGTTAGGCTAGGTGGATGCGGTAGGTCCGAATATAAGGCTgtctc                                                                                                                                                                                    >  1:562194/1‑297 (MQ=255)`  
                                                                                                                                `ggTAAGATGGGTCCTAATTGGGCGATTTGCCACTGATACAGGAGGATACTCATGAACGAAAGACACTTAACAGGTGCTGCTTCTGAAATGCTAGTAGCCTACAAATTTACCAAAGCTGGGTACACTGTCTATTACCCTATGCTGACTCAGAGTAAAGAGGACTTGGTTGTATGTAAGGATGGTAAATTTAGTAAGGTTCAGGTTAAAACAGCCACAACGGTTCAAACCAACACAGGAGATGCCAAGCAGGTTAGGCTAGGTGGATGCGGTAGGTCCGAATATAAGGATGGAGAcctgtctc                                                                                                                                                                            >  1:414751/1‑294 (MQ=255)`  
                                                                                                                                          `gTCCTAATTGGGCGATTTGCCACTGATACAGGAGGCTACTCATGAACGAAAGACACTTAACAGGTGCTGCTTCTGAAATGCTAGTAGCCTACAAATTTACCAAAGCTGGGTACACTGTCTATTACCCTATGCTGACTCAGAGTAAAGAGGACTTGGTTGTATGTAAGGATGGTAAATTTAGTAAGGTTCAGGTTAAAACAGCCACAACGGTTCAAACCAACACAGGAGATGCCAAGCAGGTTAGGCTAGGTGGATGCGGTAGGTCCGAATATAAGGATGGAGACTTTGACATTCTTGCGGt                                                                                                                                                                  <  1:41717/301‑1 (MQ=255)`  
                                                                                                                                          `cgtgggctcggagatgtgtataagagacagggaggCTACTCATGAATGAAAGACACTTAACAGGTGCTGCTTCTGAAATGCTAGTAGCCTACAAATTTACCAAAGCTGGGTACACTATCTATTACCCTATGCTGACTAAGAGTAAAGAGGACTTGGTTGTATGTAAGGATGGTAAATTTAGTAAGGTTCAGGTTAAAACAGCCACAACGGTTCAAACCAACACAGGAGATGCCAAGCAGGTTAGGCTAGGTGGATGCGGTAGGTCCGAATATAAGGATGGAGACTTTGACATTCTTGCGGt                                                                                                                                                                  <  1:495672/271‑1 (MQ=255)`  
                                                                                                                                                 `ttGGGCGATTTGCCACTGATACAGGAGGCTACTCATGAACGAAAGACACTTAACAGGTGCTGCTTCTGAAATGCTAGTAGCCTACAAATTTACCAAAGCTGGGTACACTGTCTATTACCCTATGCTGACTCAGAGTAAAGAGGACTTGGTTGTATGTAAGGATGGTAAATTTAGTAAGGTTCAGGTTAAAACAGCCACAACGGTTCAAACCAACACAGGAGATGCCAAGCAGGTTAGGCTAGGTGGATGCGGTAGGTCCGAATATAAGGATGGAGACTTTGACATTCTTGCGGTTGTGGtt                                                                                                                                                           <  1:313818/301‑1 (MQ=255)`  
                                                                                                                                                 `atgtgtataagagacaggATACAGGAGGCTACTCATGAACGAAAGACACTTAACAGGTGCTGCTTCTGAAATGCTAGTAGCCTACAAATTTACCAAAGCTGGGTACACTGTCTATTACCCTATGCTGACTCAGAGTAAAGAGGACTTGGTTGTATGTAAGGATGGTAAATTTAGTAAGGTTCAGGTTAAAACAGCCACAACGGTTCAAACCAACACAGGAGATGCCAAGCAGGTTAGGCTAGGTGGATGCGGTAGGTCCGAATATAAGGATGGAGACTTTGACATTCTTGCGGTTGTGGtt                                                                                                                                                           <  1:189763/284‑1 (MQ=255)`  
                                                                                                                                                      `ggctcggagatgtgtataagagacagcTCATTAACGAAAGACACTTAACAGGTGCTGCTTCTGAAATGCTAGTAGCCTACAAATTTACCAAAGCTGGGTACACTGTCTATTACCCTATGCTGACTCAGAGTAAAGAGGACTTGGTTGTATGTAAGGATGGTAAATTTAGTAAGGTTCAGGTTAAAACAGCCACAACGGTTCAAACCAACACAGGAGATGCCAAGCAGGTTAGGCTAGGTGGATGCGGTAGGTCCGAATATAAGGATGGAGACTTTGACATTCTTGCGGTTGTGGTTGACGa                                                                                                                                                      <  1:120571/275‑1 (MQ=255)`  
                                                                                                                                                            `gCCACTGATACAGGAGGCTACTCATGAACGAAAGACACTTAACAGGTGCTGCTTCTGAAATGCTAGTAGCCTACAAATTTACCAAAGCTGGGTACACTGTCTATTACCCTATGCTGACTCAGAGTAAAGAGGACTTGGTTGTATGTAAGGATGGTAAATTTAGTACGGTTCAGGTTAAAACAGCCACAACGGTTCAAACCAACACAGGAGATGCCAAGCAGGTTAGGCTAGGTGGATGCGGTAGGTCCGAATATAAGGATGGAGACTTTGACATTCTTGCGGTTGTGGTTGACGAAGAtgt                                                                                                                                                >  1:9294/1‑301 (MQ=255)`  
                                                                                                                                                            `gCCACTGATACAGGAGGCTACTCATGAACGAAAGACACTTAACAGGTGCCGCTTCTGAAATGCTAGTAGCCTACAAATTTACCAAAGCTGGGTACACTGTCTATTACCCTATGCTGACTCAGAGTAAAGAGGACTTGATTGTATGTAAGGATGGTAAATTTAGTAAGGTTCAGGTTAAAGCAGCCACAACGGTTCAAACCAACACAGGAGATGCCAAGCAGGTTAGGCTAGGTGGATGCGGTAGGTCCGAATATAAGGATGTAGACTTTGACATTCTTGCGGTTGTGGTTGACGAAGAtgt                                                                                                                                                >  1:323930/1‑301 (MQ=255)`  
                                                                                                                                                                `cTGATACAGGAGGCTACTCATGAACGAAAGACACTTAACAGGTGCTGCTTCTGAAATGCTAGTAGCCTACAAATTTACCAAAGCTGGGTACACTGTCTATTACCCTATGCTGACTCAGAGTAAAGATGACTTGGTTGTATGTAAGGATGGTAAATTTAGTAAGGTTCAGGTTAAAACAGCCACAACGGTTCAAACCAACACATGAGATGCCAAGCAGGTTAGGCTAGGTTGATGCGGTAGGTCCGAATATAAGGATGGAGACTTTGACATTCTTGCGGTTGTGGTTGACGAAGATGTGCtt                                                                                                                                            >  1:552620/1‑301 (MQ=255)`  
                                                                                                                                                                   `aTACAGGAGGCTACTCATGAACGAAAGACACTTAACAGGTGCTGCTTCTGAAATGCTAGTAGCCTACAAATTTACCAAAGCTGGGTACACTGTCTATTACCCTATGCTGACTCAGAGTAAAGAGGACTTGGTTGTATGTAAGGATGGTAAATTTAGTAAGGTTCAGGTTAAAACAGCCACAACGGTTCAAACCAACACAGGAGATGCCAAGCAGGTTAGGCTAGGTGGATGCGGTAGGTCCGAATATAAGGAAGGAGACTTTGACATTCTTGCGGTTGTGGTTGACGAAGATGTGCTTAtt                                                                                                                                         >  1:556730/1‑301 (MQ=255)`  
                                                                                                                                                                      `cgggaggCTACTCATGAACGAAAGACACTTAACAGGTGCTGCTTCTGAAATGCTAGTAGCCTACAAATTTACCAAAGCTGGGTACACTGTCTATTACCCTATGCTGACTCAGAGTAAAGAGGACTTGGTTGTATGTAAGGATGGTAAATTTAGTAAGGTTCAGGTTAAAACAGCCACAACGGTTCAAACCAACACAGGAGATGCCAAGCAGGTTAGGCTAGGTGGATGCGGTAGGTCCGAATATAAGGATGGAGACTTTGACATTCTTGCGGTTGTGGTTGACGAAGATGTGCTTAttttt                                                                                                                                      >  1:214884/3‑300 (MQ=255)`  
                                                                                                                                                                             `cTACTCATGAACGAAAGACACTTAACAGGTGCTGCTTCTGAAATGCTAGTAGCCTACAAATTTACCAAAGCTGGGTACACTGTCTATTACCCTATGCTGACTCAGAGTAAAGAGGACTTGGTTGTATGTAAGGATGGTAAATTTAGTAAGGTTCAGGTTAAAACAGCCACAACGGTTCAAACCAACACAGGAGATGCCAAGCAGGTTAGGCTAGGTGGATGCGGTAGGTCCGAATATAAGGATGGAGACTTTGACATTCTTGCGGTTGTGGTTGACGAAGATGTGCTTATTTTCACATggg                                                                                                                               >  1:733298/1‑301 (MQ=255)`  
                                                                                                                                                                               `aCTCATGAACGAAAGACACTTAACAGGTGCTGCTTCTGAAATGCTAGTAGCCTACAAATTTACCAAAGCTGGGTACACTGTCTATTACCCTATGCTGACTCAGAGTAAAGAGGACTTGGTTGTATGTAAGGATGGTAAATTTAGTAAGGTTCAGGTTAAAACAGCCACAACGGTTCAAACCAACACAGGAGATGCCAAGCAGGTTAGGCTAGGTGGATGCGGTAGGTCCGAATATAAGGATGGAGACTTTGACATTCTTGCGGTTGTGGTTGACGAAGATGTGCTTATTTTCACATGGGAc                                                                                                                             >  1:690345/1‑301 (MQ=255)`  
                                                                                                                                                                                `cTCATGAACGAAAGACACTTAACAGGTGCTGCTTCTGAAATGCTAGTAGCCTACAAATTTACCAAAGCTGGGTACACTGTCTATTACCCTATGCTGACTCAGAGTAAAGAGGACTTGGTTGTATGTAAGGATGGTAAATTTAGTAAGGTTCAGGTTAAAACATCCACAACGGTTCAAACCAACACAGGAGATGCCAAGCAGGTTAGGCTAGGTGGATGCGGTAGGTCCGAATATAAGGATGGAGACCTTGACATTCTTGCGGTTGTGGTTGACGAAGAtgctgtctcttatacacatctcc                                                                                                                            >  1:482897/1‑280 (MQ=255)`  
                                                                                                                                                                                    `agAACGAAAGACACTTAACAGGTGCTGCTTCTGAAATGCTAGTAGCCTACAAATTTACCAAAGCTGGGTACACTGTCTATTACCATATGCTGACTCAGAGTAAAGAGGACTTGGTTGTATGTAAGGATGGTAAATTTAGTAAGGTTCAGGTTAAAACAACCACAACGGTTCAAACCAACACAGGAGATGCCAAGCAGGTTAGGCTAGGTGGATGCGGTAGGTCCGAATATAAGGATGGAGACTTTGACATTCTTGCGGTTGTGGTTGACGAAGATGTGCTTATTTTCACATGGGACGAAGt                                                                                                                        <  1:388622/300‑1 (MQ=255)`  
                                                                                                                                                                                               `cacTTAACAGGTGCTGCTTCTGAAATGCTAGTAGCCTACAAATTTACCAAAGCTGGGTACACTGTCTATTACCCTATGCTGACTCAGAGTAAAGAGGACTTGGTTGTATGTAAGGATGGTAAATTTAGTAAGGTTCAGGTTAAAACAGCCACAACGGTTCAAACCAACACAGGAGATGCCAAGCAGGTTAGGCTAGGTGGATGCGGTAGGTCCGAATATAAGGATGGAGACTTTGACATTCTTGCGGTTGTGGTTGACGAAGATGTGCTTATTTTCACATGGGACGCAGTAAAAGGTAAGa                                                                                                             >  1:427377/1‑301 (MQ=255)`  
                                                                                                                                                                                                `acTTAACAGGTGCTGCTTCTGAAATGCTAGTAGCCTACAAATTTACCAAAGCTGGGTACACTGTCTATTACCCTATGCTGACTCAGAGTAAAGAGGACTTGGTTGTATGTAAGGATGGTAAATTTAGTAAGGTTCAGGTTAAAACAGCCACAACGGTTCAAACCAACACAGGAGATGCCAAGCAGGTTAGGCTAGGTGGATGCGGTAGGTCCGAATATAAGGATGGAGACTTTGACATTCTTGCGGTTGTGGTTGACGAAGATGTGCTTATTTTCACATGGGACGAAGTAAAAGGTAAGAc                                                                                                            <  1:609551/301‑1 (MQ=255)`  
                                                                                                                                                                                                 `cTTAACAGGTGCTGCTTCTGAAATGCTAGTAGCCTACAAATTTACCAAAGCTTGGTACACTGTCTATTACCCTATGCTGACTCAGAGTAAAGAGGACTTGGTTGTATGTAAGGATGGTAAATTTAGTAAGGTTCAGGTTAAAACAGCCACAACGGTTCAAACAAACACATGAGATGCCAAGCAGGTTAGGCTAGGTGGATGCGGTAGGTCCGAATATAAGGATGGAGACTTTGACATTCTTGCGTTTGTGGTTGACGAAGATGTGCTTATTTTCACCTgctgtctcttatacacatctccg                                                                                                           >  1:361333/1‑279 (MQ=255)`  
                                                                                                                                                                                                  `ttAACAGGTGCTGCTTCTGAAATGCTAGTAGCCTACAAATTTACCAAAGCTGGGTACACTGTCTATTACCCTATGCTGACTCAGAGTAAAGAGGACTTGGTTGTATGTAAGGATGGTAAATTTAGTAAGGTTCAGGTTAAAACAGCCACAACGGTTCAAACCAACACAGGAGATGCCAAGCAGGTTAGGCTAGGTGGATGCGGTAGGTCCGAATATAAGGATGGAGACTTTGACATTCTTGCGGTTGTGGTTGACGAAGATGTGCTTATTTTCACATGGGACGAAGTAAAAGGTAAGACAt                                                                                                          <  1:291519/301‑1 (MQ=255)`  
                                                                                                                                                                                                        `ggTGCTGCTTCTGAAATGCTAGTAGCCTACAAATTTACCAAAGCTGGGTACACTGTCTATTACCCTATGCTGACTCAGAGTAAAGAGGACTTGGTTGTATGTAAGGATGGTAAATTTAGTAAGGTTCAGGTTAAAACAGCCACAACGGTTCAAACCAACACAGGAGATGCCAAGCAGGTTAGGCTATGTGGATGCGGTAGGTCCGAATATAAGGATGGAGACTTTGACATTCTTGCGGTTGTGGTTGACGAAGATGTGCTTATTTTCACATGGGACGAAGTAAAAGGTAAGACATCCAtgt                                                                                                    <  1:41008/301‑1 (MQ=255)`  
                                                                                                                                                                                                         `gTGCTGCTTCGGAAATGCTAGTAGCCTACAAATTGACCAAAGCTGGGTACACTGTCTATTACCCTATGCTGACTCAGAGTAAAGAGGACTTGGTTGTATGTAAGGATGGTAAATTTAGTAAGGTTCAGGTTAAAACAGCCACAACGGTTCAAACCAACACAGGAGATGACAAGCAGGTTATGCTAGGTGGATGCTGTAGGTCCGAATATAAGGATGGAGACTTTGACATTCTTGCGATTGTGGTTGACGAAGATGTGCTTATTTTCACATGGGACGAAGTAAAAGGTAAGACATCCAtgtg                                                                                                   <  1:341868/301‑1 (MQ=255)`  
                                                                                                                                                                                                               `ctTCTGAAATGCTAGTAGCCTACAAATTTACCAAAGCTGGGTACACTGTCTATTACCCTATGCTGACTCAGAGTAAAGAGGACTTGGTTGTATGTAAGGATGGTAAATTTAGTAAGGTTCAGGTTAAAACAGCCACAACGGTTCAAACCAACACAGGAGATGCCAAGCAGGTTAGGCTAGGTGGATGCGGTAGGTCCGAATATAAGGATGGAGACTTTGACATTCTTGCGGTTGTGGTTGACGAAGATGTGCTTATTTTCACATTGGACGAAGTAAAAGGTAAGACATCCAtgtgtctgtc                                                                                             >  1:306986/1‑296 (MQ=255)`  
                                                                                                                                                                                                                `ttCTGAAATGCTAGTAGCCTACAAATTTACCAAAGCTGGGTACACTGTCTATTACCCTATGCTGACTCAGAGTAAAGAGGACTTGGTTGTATGTAAGGATGGTAAATTTAGTAAGGTTCAGGTTAAAACAGCCACAACGGTTCAAACCAACACAGGAGATGCCAAGCAGGTTAGGCTAGGTGGATGCGGTAGGTCCGAATATAAGGATGGAGACTTTGACATTCTTGCGGTTGTGGTTGACGAAGATGTGCTTATTTTCACATGGGACGAAGTAAAAGGTAAGACATCCATGTGTGTCGGc                                                                                            <  1:348324/301‑1 (MQ=255)`  
                                                                                                                                                                                                                 `tCTGAAATGCTAGTAGCCTACAAATTTACCAAAGCTGGGTACACTGTCTATTACCCTATGCTGACTCAGAGTAAAGAGGACTTGGTTGTATGTAAGGATGGTAAATTTAGTAAGGTTCAGGTTAAAACAGCCACAACGGTTCAAACCAACACAGGAGATGCCAAGCAGGTTAGGCTAGGTGGATGCGGTAGGTCCGAATATAAGGATGGAGACTTTGACATTCTTGCGGTTGTGGTTGACGAAGATGTGCTTATTTTCACATGGGACGAAGTAAAAGGTAAGACATCCATGTGTGTCGGCa                                                                                           <  1:107272/301‑1 (MQ=255)`  
                                                                                                                                                                                                                           `tagtagCCTACAAATTTACCAAAGCTGGGTACACTGTCTATTACCCTATGCTGACTCAGAGTAAAGAGGACTTGGTTGTATGTAAGGATGGTAAATTTAGTAAGGTTCAGGTTAAAACAGCCACAACGGTTCAAACCAACACAGGAGATGCCAAGCAGGTTAGGCTAGGTGGATGCGGTAGGTCCGAATATAAGGATGGAGACTTTGACATTCTTGCGGTTGTGGTTGACGAGGATGTGCTTATTTTCACATGGGACGAAGTAAAAGGTAAGACATCCATGTGTGTCGGCAAGAGAAACaa                                                                                 <  1:750599/301‑1 (MQ=255)`  
                                                                                                                                                                                                                                           `agatgtgtataagagacaggTCTATTACCCTATGCTGACTCAGAGTAAAGAGGACTTGGTTGTATGTAAGGATGGTAAATTTAGTAAGGTTCAGGTTAAAACAGCCACAACGGTTCAAACCAACACAGGAGATGCCAAGCAGGTTAGGCTAGGTGGATGCGGTAGGTCCGAATATAAGGATGGAGACTTTGACATTCTTGCGGTTGTGGTTGACGAAGATGTGCTTATTTTCACATGGGACGAAGTAAAAGGTAAGACATCCATGTGTGTCGGCAAGAGAAACAAAGGCATAAAACTATAg                                                                 <  1:524201/282‑1 (MQ=255)`  
                                                                                                                                                                                                                                            `gatgtgtataagagacaggTCTATTACCCTATGCTGACTCAGAGTAAAGAGGACTTGGTTGTATGTAAGGATGGTAAATTTAGTAAGGTTCAGGTTAAAACAGCCACAACGGTTCAAACCAACACAGGAGATGCCAAGCAGGTTAGGCTAGGTGGATGCGGTAGGTCCGAATATAAGGATGGAGACTTTGACATTCTTGCTGTTGTGGTTGACGAAGATGTGCTTATTTTCACATGGGACGAAGTAAAAGGTAAGACATCCATGTGTGTCGGCAAGAGAAACAAAGGCATAAAACTATAgg                                                                <  1:2019/283‑1 (MQ=255)`  
                                                                                                                                                                                                                                            `gatgtgtataagagacaggTCTATTACCCTATGCTGACTCAGAGTAAAGAGGACTTGGTTGTATGTAAGGATGGTAAATTTAGTAAGGTTCAGGTTAAAACAGCAACAACGGTTAAAACCAACACAGGAGATGCCAAGCAGGTTAGGCTAGGTGGATGCGGTAGGTCCGAATATAAGGATGGAGACTTTGACATTCTTGCGGTTGTGGTTGACGAAGATGTGCTTATTTTCACATGGGACGAAGTAAAAGGTAAGACATCCATGTGTGTCGGCAAGAGAAACAAAGGCATAAAACTATAgg                                                                <  1:594830/283‑1 (MQ=255)`  
                                                                                                                                                                                                                                              `tgtgtataagagacaggTCTATTACCCTATGCTGACTCAGAGTAAAGAGGACTTGGTTGTATGTAAGGATGGTAAATTTAGTAAGGTTCAGGTTAAAACAGCCACAACGGTTCAAACCAACACAGGAGATGCCAAGCAGGTTAGGCTAGGTGGATGCGGTAGGTCCGAATATAAGGATTGAGACTTTGACATTCTTGCGGTTGTGGTTGATGAAGATGTGCTTATTTTCACATGGGACGAAGTAAAAGGTAAGACATCCATGTGTGTTGGCAAGAGAAACAAAGGCATAAAACTATAGgag                                                              <  1:504335/285‑1 (MQ=255)`  
                                                                                                                                                                                                                                              `tgtgtataagagacaggTCTATTAAGCGATGCTGACTCAAAGTAAAGAGGACTTGGGTGGAGGTAGGGATGGTAAATTTAGTAAGGTTCAGGTTAATACAGCCACAACGGTTCAAACCAAAGCAGGAGATGAAAAGCAGGTTAGGCTAGGTGGATGCGGTAGATCCGAATATAAGGATGGAGACTTTGACATTCTTGCGGTTGTGGTTGACGAAGATGTGCTTATTTTCACATTGGACGAAGTAAAAGGTAAGACAGCCATGTGTGACGGCAAGAGAAACAAAGGCATAAAACTATAGgag                                                              <  1:11427/285‑1 (MQ=255)`  
                                                                                                                                                                                                                                              `cAAAGCTGGGTACACTGTCTATTACCATATGCTGACTCAGAGTAAAGAGGACTTGGTTGTATGTAAGGATGGTAAATTTAGTAAGGTTCAGGTTAAAACAGCCACAACGGTTCAAACCAACACAGGAGATGCCAAGCAGGTTAGGATAGGTGGATGCGGTAGGTCCGAATATAAGGATGGAGACTTTGACATTCTTGCGGTTGTGGTTGACGAAGATGTGCTTATTTTCACATGGGACGAAGTAAAAGGTAAAACATCCATGTGTGTCGGCAAGAGAAACAAAGGCATAAAACTATAGgag                                                              <  1:476377/301‑1 (MQ=255)`  
                                                                                                                                                                                                                                                   `cTGGGTACACTGTCTATTACCCTATGCTGACTCAGAGTAAAGAGGACTTGGTTGTATGTAAGGATGGTAAATTTAGTAAGGTTCAGGTTAAAACAGCCACAACGGTTCAAACCAACACAGGAGATGCCAAGCAGGTTAGGCTAGGTGGATGCGGTAGGTCCGAATATAAGGATGGAGACTTTGACATTCTTGCGATTGTGGTTGACGAAGATGTGCTTATTTTCACATGGTACGAAGTAAAAGGTAAGACATCCATGTGTGTCGGCAAGAGAAACAAAGGCATAAAACTATAggctgtctc                                                         >  1:628686/1‑294 (MQ=255)`  
                                                                                                                                                                                                                                                      `ggTACACTGTCTATTACCCTATGCTGACTCAGAGTAAAGAGGACTTGGTTGTATGTAAGGATGGTAAATTTAGTAAGGTTCAGGTTAAAACTGCCACAACGGTTCAAACCAACACATTCTATGCCAAGCCGGTTAGGCTAGTTGCTTTAGGTAGGTCCGTATATAAGGATGGAGACTTTGACATTCTTGCGGTTGTGGTTGACGAAGATGTGCTTATTTTCACATTTTACGCAGTAACAGGTAAGACATCCATGTGTGTCGGCAAGAGAAACAAAGGCATAAATCTATAGGCGAActtatt                                                      >  1:717089/1‑301 (MQ=255)`  
                                                                                                                                                                                                                                                      `ggTACACTGTCTATTACCCTATGCTGACTCAGAGTAAAGAGGACTTGGTTGTATGTAAGGATGGTAAATTTAGTAAGGTTCAGGTTAAAACAGCCACAACGGTTCAAACCAACACAGGAGATGCCAAGCAGGTTAGGCTAGGTGGATGCGGTAGGTCCGAATATAAGGATGGAGACTTTGACATTCTTGCGGTTGTGGTTGACGAAGATGTGCTTATTTTCACATGGGACGAAGTAAAAGGTAAGACATCCATGTGTGTCGGCAAGAGAAACAAAGGCATAAAACTATAGCGGAAattatt                                                      >  1:793450/1‑301 (MQ=255)`  
                                                                                                                                                                                                                                                          `ataagagacagaCCCTATGCTGACTCAGAGTAAAGAGGACTTGGTTGTATGTAAGGATGGTAAATTTAGTAAGGTTCAGGTTAAAACAGCCACAACGGTTCAAACCAACACAGGAGATGCCAAGCAGGTTAGGCTAGGTGGATGCGGTAGGTCCGAATATAAGGATGGAGACTTTGACATTCTTGCGGTTGTGGTTGACGAAGATGTGCTTATTTTCACATGGGACGAAGTAAAAGGTAAGACATCCATGTGTGTCGGCAAGAGAAACAAAGGCATAAAACTATAGGAGAAATTATTATgg                                                  <  1:81892/290‑1 (MQ=255)`  
                                                                                                                                                                                                                                                           `acagTCTATTACCATATGCTGAGTCAGAGTAAAGAGGACTTGGTTGTATGTAAGGATAGTAAATTTAGTAAGGTGCAGGTTAAAACAGCCACAACGGTTCAAACCAACACAGGAGATGCTAAGCAGATTAGGCTAGGTGGATGCGGTAGATCCGAATATAAGGATGGAGACTTTGACATTCTTGCGGTTGTGGTTGACGAAGATGTGCTTATTTTCACATGGGACGAAGTAAAATGTAAGACATCCATGTGTGTCGGCAAGAGAAACAAAGGCATAAAACTATAGGAGAAATTATTAGGGc                                                 <  1:84490/298‑1 (MQ=255)`  
                                                                                                                                                                                                                                                            `cTGTCTATTACCCTATGCTTACTCAGAGTAAAGAGGACTTGGTTGTATGTAAGGATGGTAAATTTAGTAAGGTTCAGGTTAAAACATCCACAACGGTTCAAACCAACACAGGAGATGCCAAGCAGGTTAGGCTAGGTGGATGCGGTAGGTCCGAATATAAGGATGGAGACTTTGACATTCTTGCGGTTGTGGTTGACGAAGATGTGCTTATTTTCACATGGGACGAAGTAAAAGGTAAGACATCCATGTGTGTCGGCAAGAGAAACAAAGGCATAAAACTATAGGAGAAATTATTATGGCt                                                <  1:125081/301‑1 (MQ=255)`  
                                                                                                                                                                                                                                                            `cTGTCTATTACCCTATGCTGACTCAGAGTAAAGAGGACTTGGTTGTATGTAAGGATGGTAAATTTAGTAAGGTTCAGGTTAAAACAGCCACAACGGTTAAAACCAACACAGGAGATGCCAAGCAGGTTAGGCTAGGTGGATGCAGTAGGTCCGAATATAAGGATGGAGACTTTGACATTCTTGCGGTTGTGGTTGACGAAGATGTGCTTATTTTCACATGGGACGAAGTAAAAGGTAAGACATCCATGTGTGTCGGCAAGAGAAACAAAGGCATAAAACTATAGgagctgtctcttataca                                                >  1:724297/1‑287 (MQ=255)`  
                                                                                                                                                                                                                                                              `gTCTATTACCCTATGCTGACTCAGAGTAAAGAGGACTTGGTTGTATGTAAGGATGGTAAATTTAGTAAGGTTCAGGTTAAAACAGCCACAACGGTTCAAACCAACACAGGAGATGCCACTCAGGTTAGGCTAGGTGGATGCGGTAGGTCCGAATATAAGGATGGAGACTTTGACATTCTTGCGGTTGTGGTTGACGAAGATGTTCTTATTTTCACATGGGACGAAGTAAAATGTAAGACATCCATGTGTGTCGGCAAGAGAAACAAAGGCATAAAACTATAGGAGAAATTATTATGTCTAt                                              >  1:534031/1‑301 (MQ=255)`  
                                                                                                                                                                                                                                                              `gTCTATTACCCTATGCTGACTCAGAGTAAAGAGGACTTGGTTGTATGTAAGGATGGTAAATTTAGTAAGGTTCAGGTTAAAACAGCCACAACGGTTCAAACCAACACAGGAGATGCCAAGCAGGTTAGGCTAGGTGGATGCGGTAGGTCCGAATATAAGGATGGAGACTTTGACATTCTTGCGGTTGTGGTTGACGAAGATGTGCTTATTTTCACATGGGACGAAGTAAACGGTAAGACATCCATGTGTGTCGGCAAGAGAAACAAAGGCATAAAACTATAGGAGAAATTATTATGGCTAt                                              >  1:242004/1‑301 (MQ=255)`  
                                                                                                                                                                                                                                                              `gTCTATTACCCTATGCTGACTCAGAGTAAAGAGGACTTGGTTGTATGTAAGGATGGTAAATTTAGTAAGGTTCAGGTTAAAACAGCCACAACGGTTCAAACCAACACAGGAGATGCCAAGCAGGTTAGGCTAGGTGGATGCGGTAGGTCCGAATATAAGGATGGAGACTTTGACATTCTTGCGGTTGTGGTTGACGAAGATGTGCTTATTTTCACATGGGACGAAGTAAAAGGTAAGACATCCATGTGTGTCGGCAAGAGAAACAAAGGCATAAAACTATAGgagctgtctcttatacaca                                              >  1:449453/1‑285 (MQ=255)`  
                                                                                                                                                                                                                                                              `gTCTATTACCCTATGCTGACTCAGAGTAAAGAGGACTTGGTTGTATGTAAGGATGGTAAATTTAGTAAGGTTCAGGTTAAAACAGCCACAACGGTTCAAACCAACACAGGAGATGCCAAGCAGGTTAGGCTAGGTGGATGCGGTAGGTCCGAATATAAGGATGGAGACTTTGACATTCTTGCGGTTGTGGTTGACGAAGATGTGCTTATTTTCACATGGGACGAAGTAAAAGGTAAGACATCCATGTGTGTCGGCAAGAGAAACAAAGGCATAAAACTATAGGAGAAATTATTATGGCTAt                                              >  1:355493/1‑301 (MQ=255)`  
                                                                                                                                                                                                                                                              `gTCTATTACCCTATGCTGACTCAGAGTAAAGAGGACTTGGTTGTATGTAAGGATGGTAAATTTAGTAAGGTTCAGGTTAAAACAGCCACAACGGTTCAAACCAACACAGGAGATGCCAAGCAGGTTAGGCTAGGTGGATGCGGTAGGTCCGAATATAAGGATGGAGACTTTGACATTCTTGCGGTTGTGGTTGACGAAGATGTGCTTATTTTCACATGGGACGAAGTAAAAGGTAAGACATCCATGTGTGTCGGCAAGAGAAACAAAGGCATAAAACTATAGGAGAAATTATTATGGCTAt                                              >  1:55004/1‑301 (MQ=255)`  
                                                                                                                                                                                                                                                              `gTCTATTACCCTATGCTGACTCAGAGTAAAGAGGACTTGGTTGTATGTAAGGATGGTAAATTTAGTAAGGTTCAGGTTAAAACAGCCACAACGGTTCAAACCAACACAGGAGATACCAAGCAGGTTAGGCTAGGTGGATGCGGTAGGTCCGAATATAAGGATGGAGACTTTGACATTCTTGCGGTTGTGGTTGACGAAGATGTGCTTATTTTCACATGGGACGAAGTAAAAGGTAAGACATCCATGTGTGTCGGCACGAGAAACAAAGGCATAAAACTATAGGAGACATTATTATGGCTAt                                              >  1:567015/1‑301 (MQ=255)`  
                                                                                                                                                                                                                                                              `gTCTATTACCCTATGCTGACTCAGAGTAAAGAGGACTTGGTTGTATGTAAGGATGGTAAATTTAGTAAGGTTCAGGTTAAAACAGCCACAACGGTTCAAACAAACACAGGAGATGCCAAGCAGGTTAGGCTAGGTGGATGCGGTAGGTCCGAATATAAGGATGGAGACTTTGACATTCTTGCGGTTGTGGTTGACGAAGATGTGCTTATTTTCACATGGGACGAAGTAAAAGGTAAGACATCCATGTGTGTCGGCAAGAGAAACAAAGGCATAAAACTATAGGAGAAATTATTATGGCTAt                                              >  1:600958/1‑301 (MQ=255)`  
                                                                                                                                                                                                                                                                `cTATTACCCTATGCTGACTCAGAGTAAAGAGGACTTGGTTGTATGTACGGATGGTAAATTTAGTAATGTTCAGGTTAAAACAGCCACAACGGTTCAAACCAACACAGGAGATGCCATGCAGGTTAGGCTAGGTGGATGCGGTAGGTCCGAATATAAGGATGGAGACTTTGACATTCTTGCGGTTGTGGTTGACGAAGATGTGCTTATTTTCACATGGGACGAAGTAAAAGGTAAGACATCCATGTGTGTCGGCAAGAGAAACAAAGGCATAAAAcctgtctcttatacacatctccgagcc                                            >  1:377864/1‑275 (MQ=255)`  
                                                                                                                                                                                                                                                                `cTATTACCCTATGCTGACTCAGAGTAAAGAGGACTTGGTTGTATGTAAGGATGGTAAATTTAGTAAGGTTCAGGTTAAAACAGCCACAACGGTTCAAACCAACACAGGAGATGCCAAGCAGGTTAGGCTAGGTGGATGCGGTAGGTCCGAATATAAGGATGGAGACTTTGACATTCTTGCGGTTGTGGTTGACGAAGCTGTGCTTAATTTCACATGGGACTAAGTAAAAGGTAAGACATCCATGTGTGTCGGCAAGAGAAACAAAGGCATAAAACTATAGGAGAAATTATTATGGCTATGa                                            >  1:163316/1‑301 (MQ=255)`  
                                                                                                                                                                                                                                                                `cTATTACCCTATGCTGACTCAGAGTAAAGAGGACTTGGTTGTATGTAAGGATAGTAAATTTAGTAAGGTTCAGGTTAAAACAGCCACAACGGTTCAAACCAACACAGGAGATGCCAAGCAGGTTAGGCTAGGTGGATGCGGTAGGTCCGAATATAAGGATGGAGACTTTGACATTCTTGCGGTTGTGGTTGACGAAGATGTGCTTATTTTCACATGGGACGAAGTAAAAGGTAAGACATCCATGTGTGTCGGCAAGAGAAACAAAGGCATAAAAcctgtctcttatacacatctccgagcc                                            >  1:370315/1‑275 (MQ=255)`  
                                                                                                                                                                                                                                                                          `aTGCTGACTCAGAGTAAAGAGGACTTGGTTTTATGTAAGGATGGTAAATTTAGTAAGGTTCAGGTTAAAACAGCCACAACGGTTCAAACCAACACAGGAGATGCCAAGCAGGTTAGGCTAGGTGGATGCGGTAGGTCCGAATATAAGGATGGAGACTTTGACATTCTTGCGGTTGTTGTTGACGAAGATGTGCTTATTTTCACATGGGACGAAGTAAAAGGTAAGACATCCATGTGTGTCGGCAAGAGAAACAAAGGCATAAAACTATAGGAGAAATTATTATGGCtggctcttatacaca                                  >  1:257307/1‑287 (MQ=255)`  
                                                                                                                                                                                                                                                                            `gCTGACTCAGAGTAAAGAGGACTTGGTTGTATGTAAGGATGGTAAATTTAGTAAGGTTCAGGTTAAAACAGCCACAACGGTTCAAACCAACACAGGAGATGCCAAGCAGGTTAGGCTAGGTGGATGCGGTAGGTCCGAATATAAGGATGGAGACTTTGACATTCTTGCGGTTGTGGTTGACGAAGATGTGCTTATTTTCACATGGGACGAAGTAAAAGGTAAGACATACATGTGTGTCGGAAAGAGAAACAAAGGCATAACACTATAGGAGAAATTATTATGGCTATGACAAAGAAATTTa                                >  1:706079/1‑301 (MQ=255)`  
                                                                                                                                                                                                                                                                               `gACTCAGAGTAAAGAGGACTTGGTTGTATGTAAGGATGGTAAATTTAGTAAGGTTCAGGTTAAAACAGCCACAACGGTTCAAACCAACACAGGAGATGCCAAGCAGGTTAGGCTAGGTGGATGCGGTAGGTCCGAATATAAGGATGGAGACTTTGACATTCTTGCGGTTGTGGTTGACGAAGATGTGCTTATTTTCACATGGGACGAAGTAAAAGGTAAGACATCCATGTGTGTCGGCAAGAGAAACAAAGGCATAAAACTATAGGAGAAATTATTATGGCTATGACAAAGAAATTTAAAg                             <  1:649975/301‑1 (MQ=255)`  
                                                                                                                                                                                                                                                                                    `agagTAAAGAGGACTTGGTTGTATGTAATGATGGTAAATTTAGTAAGGTTCAGGTTAAAACAGCCACAACGGTTCAAACCAACACAGGAGATGCCAAGCAGGTTAGGCTAGGTGGATGCGGTAGGTCCGAATATAATGATGGAGACTTTGACATTCTTGCGGTTGTGGTTGACGAAGATGTGCTTATTTTCACATGGGACGAAGTAAAAGGTAAGACATCCATGTGTGTCGGCAAGAGAAACAAAGGCATAAAACTATAGGAGCAATTATTATGGCTATGACAAAGAAATTTAAAGTGTcc                        >  1:457399/1‑301 (MQ=255)`  
                                                                                                                                                                                                                                                                                     `gagTAAAGAGGACTTGGTTGTATGTAAGGATGGTAAATTTAGTACGGTTCAGGTTAAAACAGCAACAACGGTTCAATACAACACAGGAGATGCCAAGCAGGTTAGGCTAGGTGGATGCGGTAGGTCCGAATATATGGATAGCGACTTTTACACTCTTACTGTTGTGGTTGACGAAGTTGTGATTATTTACAAATGGTACGAAGTAAACGATAAGACATCAATTTTTGTCGGCATGAGAAACAAATGCATAAAAATATCGGAGATATACTTCTTGCTATGACTAAGAAATTTAAATTGTTCt                       >  1:823706/1‑301 (MQ=255)`  
                                                                                                                                                                                                                                                                                     `gagTAAAGAGGACTTGGTTGTATGTAAGGATGGTAAATTTAGTAAGGTTCAGGTTAAAACAGCCACAACGGTTCAAACCAACACAGGAGATGCCAAGCAGGTTAGGCTAGGTGGATGCGGTAGGTCCGAATATAAGGATGGAGACTTTGACATTCTTGCGGTTGTGGTTGACGAAGATGTGCTTATTTTCACATGGGACGAAGTAAAAGGTAAGACATCCATGTGTGTCGGCAAGAGAAACAAAGGCATAAAACTATAGGAGAACTTATTATGGCTATGACAAAGGAATTTAAAGTGTCCt                       >  1:385352/1‑301 (MQ=255)`  
                                                                                                                                                                                                                                                                                     `gagTAAAGAGGACTTGGTTGTATGTAAGGATGGTAAATTTAGTAAGGTTCAGGTTAAAACAGCCACAACGGTTCAAACCAACACAGGAGATGCCAAGCAGGTTAGGCTAGGTGGATGCGGTAGGTCCGAATATAAGGATGGAGACTTTGACATTCTTGCGGTTGTGGTTGACGAAGATGTGCTTATTTTCACATGGGACGAAGTAAAAGGTAAGACATACATGTGTGTCGGCAAGAGAAACAAAGGCATAAAAATATAGGAGAAATTATTATGGCTATGACAAAGAAATTTAAAGTGTcat                       >  1:793273/1‑299 (MQ=255)`  
                                                                                                                                                                                                                                                                                       `gTAAAGAGGACTTGGTTGTATGTAAGGATGGTAAATTGAGTAAGGTTCAGGTTAAAACAGCCACAACGGTTCAAACCAACACAGGAGATGCCAAGCAGGTTAGGCTAGGTGGATGCGGTAGGTCCGAATATAAGGATGGAGACGTTGACATGCTTGCGGTTGTGGTTGACGAAGATGTGCTGATTTTCACATGGGACGAAGTAAAAGGTAAGACATCCATGTGTGTCGGCAAGAGAAGCAAAGGCATAAAACTATAGGAGAAATTATTATGGCTATGACAAAGAAATTTAAAGTGTCCTTc                     <  1:363604/301‑1 (MQ=255)`  
                                                                                                                                                                                                                                                                                         `aaaGAGGACTTGGTTGTATGTAAGGATGGTAAATTTAGTAAGGTTCAGGTTAAAACAGCCACAACGGTTCAAACCAACACAGGAGATGCCAAGCAGGTTAGGCTAGGTGGATGCGGTAGGTCCGAATATAAGGATGGAGACTTTGACATTCTTGCGGTTGTGGTTGACGAAGATGTGCTTATTTTCACATGGGACGAAGTAAAAGGTAAGACATCCATGTGTGTCGGCAAGAGAAACAAGGGCATAAAACTATAGGAGAAATTATTATGGCTATGACAAAGAAATTTAAAGTGTCCTTCGa                   >  1:509114/1‑301 (MQ=255)`  
                                                                                                                                                                                                                                                                                         `aaaGAGGACTTGGTTGTACGTAAGGATGGTAAATTTAGTAAGGTCCAGGTTAAAACAGCCACAACGGTTCAAACCAACACAGGAGATGCCAAGCAGGTTAGGCTAGGTGGATGCGGTAGGTCCGAATATAAGGATGGAGACTTTGACATTCTTGCGGTTGTGGTTGACGAAGATGTGCTTATTTTCACATGGGACGAAGTAAAAGGTAAGACATCCATGTGTGTCGGCAAGAGAAACAAAGGCATAAAACTATAGGAGAAATTATTATGGCtgtctcttatacacatctccgagcccacga                   >  1:382023/1‑272 (MQ=255)`  
                                                                                                                                                                                                                                                                                          `tgtgtagaagagacagatgtaAGGATGGTAAATTTAGTAAGGTTCAGGTTAAAACAGCCACAACGGTTCAAACCAACACAGGAGATGCCAAGCAGGTTAGGCTAGGTGGATGCGGTAGGTCCGAATATAAGGATGGAGACTTTGACATTCTTGCGGTTGTGGTCGACGAAGATGTGCTTATTTTCACATGGGACAAAGTAAAAGGTAAGACATCCATGTGTGTCGGCAAGAGAAACAAAGGCATAAAACTATAGGAGAAATTATTATGGCTATGACAAAGAAATTTAAAGTGTCCTTCGAc                  <  1:332182/285‑1 (MQ=255)`  
                                                                                                                                                                                                                                                                                                `aCTTGGTTGTATGTAAGGATGGTAAATTTAGTAAGGTTCAGGTTAAAACAGCCACAACGGTTCAAACCAACACAGGAGATGCCCAGCAGGTTAGGATAGGTGGATGCGGTAGGTCCGAATATAAGGATGGAGACTTTGACATTCTTGCGGTTGTGGTTGACGAAGATGTGCTTATTTTCACATGTGACGAAGTAAAAGGTAAGACATCCATGTGTGTCGGCAAGAGAAACAAAGGCATACAGCTCTAGGAGCACTTATTATGGCTATGACAAAGAACTTTAAAGTGTCCTTCGACCTTAcc            >  1:764846/1‑301 (MQ=255)`  
                                                                                                                                                                                                                                                                                                      `aagtatgtaAGGATGGTAAATTTAGTAAGGTTCATGTTAAAACAGCCACAACGGTTCAAACCAACACAGGAGATGCCAAGCAGGTTAGGCTAGGTGGATGCGGTAGGTCCGAATATAAGGATGGAGACTTTGACATTCTTGCGGTTGTGGTTGACGAAGATGTGCTTATTTTCACATGGGACGAAGTAAAAGGTAAGACATCCATGTGTGTCGGCAAGAGAAACAAAGGCATAAAACTATATGAGAAATTATTATGGCTATGACAAAGAAATTTAAAGTGTCCTTCGACGTTACCGCAAAg      <  1:738480/299‑1 (MQ=255)`  
                                                                                                                                                                                                                                                                                                         `tatgtaAGGATGGTAAATTTAGTAAGGTTCAGGTTAAAACAGCCACAACGGTTCAAACCAACACAGGAGATGCCAAGCAGGTTAGGCTAGGTGGATGCGGTAGGTCCGAATATAAGGATGGAGACTTTGACATTCTTGCGGTTGTGGTTGACGAAGATGTGCTTATTTTCACATGGGACGAAGTAAAAGGTAAGACATCCATGTGTGTCGGCAAGAGAAACAAAGGCATAAAACTATAGGAGAAATTATTATGGCTATGACAAAGAAATTTAAAGTGTCCTTCGACG‑TACCGCAAAGATgt  >  1:110205/1‑301 (MQ=255)`  
                                                                                                                                                                                                                                                                                                          `atgtaAGGATTGTAAATTTAGTAAGGTTCAGGTTAAAACAGCCACAACGGTTCAAACCAACACAGGAGATGCCAAGCAGGTTAGGCTAGGTGGATGTGGTAGGTCCGAATATAAGGATGGAGACTTTGACATTCTTGCGGTTGTGGTTGACGAAGATGTGCTTATTTTCACATGGGACGAAGTAAAAGGTAAGACATCCATGTGTGTCGGCAAGAGAAACAAAGGCATAAAACTATAGTAGAAATTATTATGGCTATGACAAAGAAATTTAAAGTGTCCTTCGACGTTACCGCAAAGATgt  >  1:660668/1‑301 (MQ=255)`  

                                                                                                                                                                                                                                                                                                            `|`                                                                                                                                                                                                                                                                                                            
`GGGTACATGATGAAATCCAAGTAGGCTGCCGTACCGAAGAGATTGCTCAGGTGGTCATTGAGACCGCACAAGAAGCGATGCGCTGGGTTGGAGACCACTGGAACTTCCGGTGTCTTCTGGATACCGAAGGTAAGATGGGTCCTAATTGGGCGATTTGCCACTGATACAGGAGGCTACTCATGAACGAAAGACACTTAACAGGTGCTGCTTCTGAAATGCTAGTAGCCTACAAATTTACCAAAGCTGGGTACACTGTCTATTACCCTATGCTGACTCAGAGTAAAGAGGACTTGGTTGTATGTAAGGATGGTAAATTTAGTAAGGTTCAGGTTAAAACAGCCACAACGGTTCAAACCAACACAGGAGATGCCAAGCAGGTTAGGCTAGGTGGATGCGGTAGGTCCGAATATAAGGATGGAGACTTTGACATTCTTGCGGTTGTGGTTGACGAAGATGTGCTTATTTTCACATGGGACGAAGTAAAAGGTAAGACATCCATGTGTGTCGGCAAGAGAAACAAAGGCATAAAACTATAGGAGAAATTATTATGGCTATGACAAAGAAATTTAAAGTGTCCTTCGACGTTACCGCAAAGATGT  >  NC_001604.1/16304‑16902`

  

Alignment Legend

Aligned base mismatch/match (shaded by quality score): `ATCG/ATCG` `< 3 ≤` `ATCG/ATCG` `< 11 ≤` `ATCG/ATCG` `< 26 ≤` `ATCG/ATCG` `< 36 ≤` `ATCG/ATCG` `< 38 ≤` `ATCG/ATCG`

Unaligned base: `atcg`    Masked matching base: `atcg`    Alignment gap: `‑`    Deleted base: `‑`

Reads not counted as support for junction

read\_name Not counted due to insufficient overlap past the breakpoint.

read\_name Not counted due to not crossing MOB target site duplication.
